# Liang-Ge decoction ameliorates acute lung injury in septic model rats through reducing inflammatory response, oxidative stress, apoptosis, and modulating host metabolism

**DOI:** 10.3389/fphar.2022.926134

**Published:** 2022-09-16

**Authors:** Wenju He, Qiang Xi, Huantian Cui, Pingping Zhang, Rui Huang, Taihuan Wang, Dongqiang Wang

**Affiliations:** ^1^ Department of Integration of Traditional Chinese and Western Medicine, First Central Hospital Affiliated to Nankai University, Tianjin First Central Hospital, Tianjin, China; ^2^ Department of Practice and Education, Tianjin University of Traditional Chinese Medicine, Tianjin, China; ^3^ Shandong Provincial Key Laboratory of Animal Cell and Developmental Biology, School of Life Sciences, Shandong University, Qingdao, China; ^4^ Department of Graduate School, Tianjin University of Traditional Chinese Medicine, Tianjin, China

**Keywords:** liang-Ge decoction, sepsis-associated lung injury, inflammatory response, oxidative stress, apoptosis, untargeted metabolomics

## Abstract

Liang-Ge decoction (LG) has been used in the treatment of early stage of spesis and can ameliorate sepsis-associated lung injury. However, the mechanism of LG on sepsis-associated lung injury remains unknown. In this study, we established a rat model of sepsis-associated lung injury using the cecal ligation and puncture (CLP) method, and investigated the therapeutic effects of LG on lung injury in rats with sepsis. In addition, the anti-inflammatory, anti-oxidative and anti-apoptotic effects of LG on sepsis-associated lung injury model rats were evaluated. Besides, untargeted metabolomics was used to investigate the regulation of metabolites in rats with sepsis-associated lung injury after LG treatment. Our results showed that LG could decrease the wet/dry (W/D) ratio in lung and the total cell count and total protein concentration in bronchoalveolar lavage fluid (BALF) in septic model rats. Hematoxylin and eosin (HE) staining showed that LG reduced the infiltration of pro-inflammatory cells in lung. In addition, LG treatmment down-regulated the gene and protein expression of pro-inflammatory cytokins in lung tissue and BALF. The activities of superoxide dismutase (SOD) and glutathione peroxidase (GSH-Px) were increased and the level of methane dicarboxylic aldehyde (MDA) was decreased in lung tissue homogenate in septic model rats after LG treament. Moreover, the numbers of apoptotic cells in lung were reduced and the activity of lactic dehydrogenase (LDH) in BALF was decreased in septic model rats after LG treament. Untargeted metabolomics analysis showed that LG treatment affected the levels of 23 metabolites in lung in septic model rats such as citric acid, methionine, threonine, alpha-ketoglutaric acid, and inositol, these metabolites were associated with the glycine, serine and threonine metabolism, cysteine and methionine metabolism, inositol phosphate metabolism and citrate cycle (TCA cycle) pathways. In conclusion, our study demonstrated the therapeutic effetcts of LG on sepsis-associated lung injury model rats. Moreover, LG could inhibit the inflammatory response, oxidative stress, apoptosis and regulate metabolites related to glycine, serine and threonine metabolism, cysteine and methionine metabolism, inositol phosphate metabolism and TCA cycle in lung in sepsis-associated lung injury model rats.

## Introduction

Sepsis is a systemic disease caused by infection that can involve numerous organs, such as the lung, kidney, heart, brain, and gastrointestinal (GI) tract. Besides, the consequent multiple organ dysfunction syndromes are the main cause of death in patients with sepsis (Xia et al., 2021). During the progression of sepsis, lung is the first and most common organ to be damaged (Wang et al., 2019; Xu et al., 2020). If patients do not receive timely and effective treatment, their condition often deteriorates rapidly into severe respiratory failure (Johnson and Matthay, 2010), which can be life threatening. Studies have shown that 40%–68.2% of adults with sepsis are accompanied by lung injury (Cecconi et al., 2018). With the widespread use of antibiotics, which has resulted in widespread drug resistance, the treatment of sepsis-associated lung injury is becoming increasingly challenging (Johnson and Matthay, 2010). Therefore, it is crucial to develop practical interventions to improve sepsis-associated lung injury.

Many studies have demonstrated the protective effects of traditional Chinese medicine (TCM) on sepsis-associated lung injury. Qing-Wen-Bai-Du decoction could improve acute lung injury and ameliorate the coagulation disorders in septic model rats (Jia et al., 2022). Xue-Bi-Jing injection could ameliorate acute lung injury in septic model rats through improving lung permeability and inhibiting inflammatory response (Liu et al., 2014). Si-Ni decoction could alleviate lung injury in cecal ligation and puncture (CLP)-induced septic model mice through modulating the gut microbiota (Wang et al., 2020). Ba-Bao-Dan treatment reduced the inflammatory response in septic model mice through inhibiting the activation of NOD-like receptor protein 3 (NLRP3)-mediated inflammasome (Li et al., 2022). Illustrating the protective mechanisms of TCM on sepsis-associated lung injury can contribute to the modernization of TCM.

Alterations in the levels of metabolites have been closely correlated with several pathological states, such as inflammation, tissue injury, apoptosis, and oxidative stress. With the use of untargeted metabolomics, it is possible to study the changes in metabolite levels during disease development. It was found that similar alterations in metabolic levels also occurred during sepsis development. In patients with sepsis, the serum levels of glucose, glycine, 3-hydroxybutyrate, creatinine, and glycoprotein acetyls are significantly increased, whereas those of citrate and histidine are significantly decreased compared with healthy subjects (Jaurila et al., 2020). Other studies have also demonstrated a correlation between the total glutathione, adenosine, phosphatidylserine, and sphingomyelin levels, and the development of sepsis-associated lung injury (Stringer et al., 2011). Moreover, adipose tissue-derived mesenchymal stem cells can suppress the inflammatory responses in the lungs of septic rats by regulating the levels of acetylcholine, spermine, phenylalanine, and threonine (Cui J. et al., 2020). Furthermore, mangiferin can improve lung injury caused by sepsis by inhibiting oxidative stress and regulating the lipid metabolism and energy biosynthesis (Wang et al., 2018; Xu et al., 2020).

Liang-Ge decoction (LG) is composed of *Rheum palmatum* L. [Polygonaceae; Rhei radix et rhizoma], *Citrullus lanatus* (Thunb.) Matsum. and Nakai [Cucurbitaceae; Mirabilitum praeparatum], *Glycyrrhiza glabra* L. [Fabaceae; Glycyrrhizae radix et rhizoma], *Gardenia jasminoides* J. Ellis [Rubiaceae; Gardeniae fructus praeparatus], *Mentha canadensis* L. [Lamiaceae; Menthae haplocalycis herba], *Scutellaria baicalensis* Georgi [Lamiaceae; Scutellariae radix], *Forsythia suspensa* (Thunb). Vahl [Oleaceae; Forsythiae fructus], and *Lophatherum gracile* Brongn. [Poaceae; Lophatheri herba], and can be used for treating the early stage of sepsis. Clinical study revealed that combination treatment of LG and western therapy could significantly suppress the inflammatory response and reduce platelet activation/thrombocytopenia in spetic petients (Wang et al., 2011). A study has demonstrated that LG can inhibit the inflammatory response in septic model mice (Yang et al., 2019); however, the mechanism of action of LG to improve the symptoms of sepsis-associated lung injury remains unclear. In this study, we established a rat model of sepsis-associated lung injury using the CLP method, and investigated the therapeutic effects of LG on lung injury in rats with sepsis. In addition, the anti-inflammatory, anti-oxidative and anti-apoptotic effects of LG on sepsis-associated lung injury model rats were evaluated. Besides, untargeted metabolomics was used to investigate the regulation of metabolites in rats with sepsis-associated lung injury after LG treatment.

## Material and methods

### Reagents

Terminal deoxynucleotidyl transferase deoxyuridine triphosphate (dUTP) nick end labeling (TUNEL) staining kit was purchased from Kaiji Biotechnology Co., Ltd. (Jiangsu, China). Enzyme-linked immunosorbent assay (ELISA) kits of rat interleukin (IL)-1β, IL-6 and umor necrosis factor alpha (TNF-α) were obtained from Shanghai Bluegene Biotech Co., Ltd. (Shanghai, China). Total protein, superoxide dismutase (SOD), methane dicarboxylic aldehyde (MDA), glutathione peroxidase (GSH-Px) and lactic dehydrogenase (LDH) assay kits were purchased from Nanjing Jiancheng Biological Engineering Institute (Nanjing, China). RNA extraction, first-strand cDNA reverse transcription, polymerase chain reaction (PCR) kits and primers were purchased from TianGen Biotechnology Co., Ltd. (Beijing, China).

### Laboratory animals

Seventy-five male Sprague Dawley rats weighing 200 ± 20 g each were obtained from Beijing Huafukang Biotechnology Co., Ltd. They were acclimatized in a quiet environment for one week with a relative humidity of 55%–60%. Adequate clean water was provided to the animals, and they were fed freely. The experiment was approved by the Ethics Committee of Tianjin First Central Hospital.

### Generation of a septic rat model

An animal model of sepsis was generated using the CLP method (Dejager et al., 2011). A 12-h fast for food and water was performed before surgery. The skin of the anterior mid-abdomen of the rats was shaved after the application of anesthesia with isoflurane gas. This skin was then disinfected and a 2-cm incision was made in the mid-abdomen. After removing the cecum, the mesentery and cecum were freed, and the end of the cecum was ligated at a site 5 mm away from the ileocecal region using a 3–0 surgical suture; then, a syringe needle (20 G) was used to perform a through-hole puncture twice at a site located approximately 0.8–1 cm away from the intestinal wall on both sides of the distal appendiceal root of the ligature, from where a small amount of intestinal content was squeezed out. Subsequently, a 3 mm× 0.5 mm× 30 mm surgical drain was placed in the cecum through-hole puncture, after which the cecum was placed back into the abdominal cavity and the incision was sutured.

### Preparation of Liang-Ge decoction

LG was firstly recorded in “Prescriptions People’s Welfare Pharmacy” in Song Dynasty and was prepared based on the description in “Prescriptions People’s Welfare Pharmacy.” Briefly, 6 g of *R. palmatum* L. [Polygonaceae; Rhei radix et rhizoma] (Batch No. 210115), 10 g of *Citrullus lanatus* (Thunb.) Matsum. and Nakai [Cucurbitaceae; Mirabilitum praeparatum] (Batch No. 210312), 6 g of *G. glabra* L. [Fabaceae; Glycyrrhizae radix et rhizoma] (Batch No. 210327), 10 g of *G. jasminoides* J. Ellis [Rubiaceae; Gardeniae fructus praeparatus] (Batch No. 211203), 6 g of *M. canadensis* L. [Lamiaceae; Menthae haplocalycis herba] (Batch No. 201127), 10 g of *S. baicalensis* Georgi [Lamiaceae; Scutellariae radix] (Batch No. 210708), 20 g of *F. suspensa* (Thunb). Vahl [Oleaceae; Forsythiae fructus] (Batch No. 210319), and 10 g of *L. gracile* Brongn. [Poaceae; Lophatheri herba] (Batch No. 210521) were weighed and soaked with an eight-fold volume of water (624 ml) for 30 min. The mixture was decocted for 30 min and filtered. The filtrate was stored separately. The dregs were then mixed with eight-fold volume of water (624 ml) for the second extraction. About 150 ml of filtrates each time were obtained and combined. Then, the combined filtrates (300 ml) were evaporated to about 50 ml (1.59 g of crude herb/ml). Then, the evaporated LG extraction was the diluted into 0.39 g/ml, and 0.78 g/ml, respectively. All herbs were obtained from Tianjin traditional Chinese Medicine prepared pieces Co., Ltd. and authenticated by pharmacist of the Tianjin First Central Hospital. The voucher specimen of herbs were deposited at the Department of Integration of Traditional Chinese and Western Medicine in Tianjin First Central Hospital.

Quality control of LG was performed using Ultra performance liquid chromatography (UPLC; ACQUITY UPLC^®^, United States) coupled with Xevo G2 quadrupole-timeof-flight (Q-TOF) mass spectrometer (MS; Waters Corp.Milford, MA, United States) systems based on our previous established protocol (Xie et al., 2021).

### Experimental groups and drug administration protocol

**FIGURE 1:**
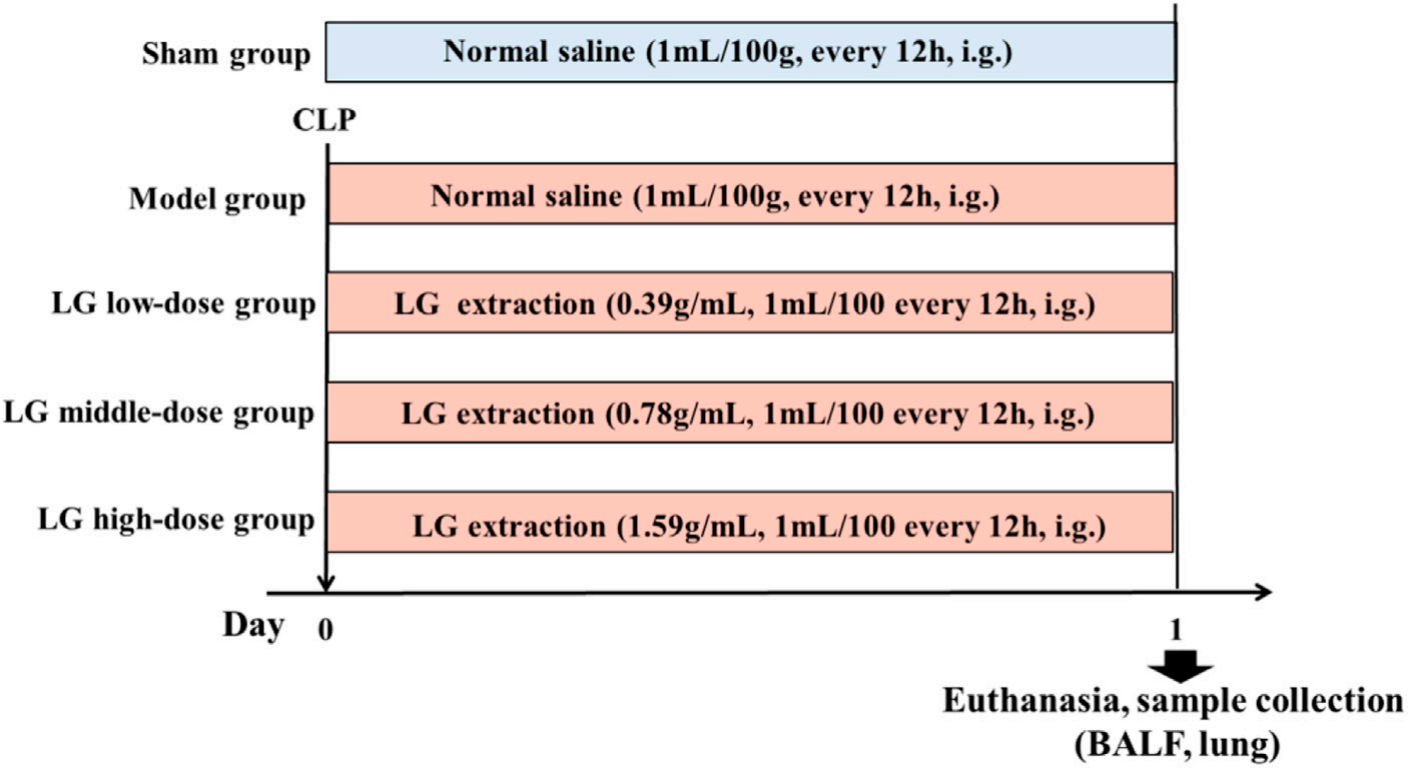
Grouping and dosing regimen. LG: Liang-Ge decoction, BALF: bronchoalveolar lavage fluid.

Seventy-five rats were selected and randomly and equally divided into the following groups: Sham, model, LG low-dose, LG middle-dose, and LG high-dose groups. Rats from model, LG low-dose, LG middle-dose, and LG high-dose groups received CLP to establish the sepsis model. In contrast, rats in the sham group only had their abdominal wall incised and sutured without receiving CLP. After modeling, the rats in the Sham and model groups were administered with 1 ml/100 g of saline intragastrically, and the rats in the LG low-, middle-, and high-dose groups were administered with 0.39 g/ml, 0.78 g/ml, and 1.59 g/ml of LG extractions every 12 h, respectively (Figure 1). The gavage amount of LG extraction was 1 ml/100 g. The dosage of LG for rats was calculated using the animal dose conversion formula based on the daily human dose, with a conversion coefficient of 6. The amount used for the middle-dose LG rats group represents the human equivalent dose using the following formula: middle-dose LG = 78 g (the total raw materials)/60 kg (human weight) × 6 (conversion coefficient).

### Collection of bronchoalveolar lavage fluid bronchoalveolar lavage fluid

After 24 h of drug administration, the thoracic cavity of the rat was opened and the cervical trachea was exposed layer by layer. A Lanz incision was made on the trachea of the rats, and a rat lavage needle was inserted into the lower end of the right main bronchus. The trachea and rat lavage needle were ligated using surgical sutures. Then, the left hilus of the rat’s lung was firmly ligated with surgical sutures, to ensure that the left lung was in an airtight state. Using a syringe, 5 ml of saline was withdrawn and connected to the lavage needle that was ligated in the cervical trachea, and saline was slowly injected into the right lung of the rats. Saline was left in the alveoli for 15–30 s and then gently sucked back to withdraw the BALF; the saline was closely observed for exudation during the lavage. This injection and withdrawal procedure was repeated thrice, and a total of approximately 10 ml of BALF was withdrawn from each rat.

### Lung wet-to-dry wet/dry weight ratio

Twenty-four hours after model generation and drug administration, the left lung of the rats, which had not been lavaged, was collected after collecting BALF from each group. The wet mass (W) of the left lung was weighed, and the organ was dried in a constant temperature incubator at 80°C for 48 h, until the lung weight no longer decreased; the lung was then weighed and the value was used as the lung dry weight (D). The W/D ratio of lung tissue was then calculated.

### Total cell count and protein concentration assay of the bronchoalveolar lavage fluid

The BALF was centrifuged at 3,000 rpm/min at 4°C for 10 min, and the cell precipitate and supernatant were collected separately. The total cell count in the BALF precipitate was assessed using a cell-counting plate. The total protein concentration in the BALF supernatant was measured using a bicinchoninic acid (BCA) total protein concentration assay kit.

### Pathological staining

Twenty-four hours after model generation and drug administration, the rats were sacrificed and the lung tissues of each group were collected, fixed in a formalin solution, embedded in paraffin, cut into 3-μm sections, routinely stained with hematoxylin and eosin (HE), and sealed with neutral balsam. The pathological changes in each group of rats were observed under a light microscope. The inflammation score of HE staining was evaluated baed on the previous study (Cui Y. et al., 2020). In addition, the level of apoptosis in lung tissue was observed using terminal deoxynucleotidyl transferase deoxyuridine triphosphate (dUTP) nick end labeling (TUNEL) staining. The positive expression area was quantified using Image-Pro Plus 6.0, and the TUNEL positive area was calculated based on the integrated optical density (IOD) and total area using the following formula: TUNEL positive area (%) = IOD/total area *100%

### Enzyme-linked immunosorbent assay

The levels of the inflammatory cytokines IL-6, IL-1β, and TNF-α in the BALF supernatant were measured based on the instructions of the ELISA kits.

### Quantitative polymerase chain reaction

Total RNA was extracted from the stored frozen lung tissues using an RNA extraction kit (TianGen Biotechnology Co., Ltd. Beijing, China). After testing the purity and concentration of the RNA, it was reverse transcribed into cDNA and used in real-time PCR to detect the expression of the *IL-6*, *IL-1β*, and *TNF-α* mRNAs in liver tissues. The relative expression of the mRNAs was calculated based on the 2^-△△CT^ quantification method using *β-actin* as a loading control. The primer sequences were designed by TianGen Biotechnology Co., Ltd. Beijing, China (Table 1).

**TABLE 1:** Primer sequences of target genes for rats.

Genes	Primer sequence (5′-3′)
β-actin	Forward: CTT​CCA​GAC​ACG​CCA​TCA​TG
	Reverse: TGG​TGA​TGG​CGT​AGA​ACA​GT
IL-1β	Forward: GGG​ATG​ATG​ACG​ACC​TGC​TA
	Reverse: TGT​CGT​TGC​TTG​TCT​CTC​CT
IL-6	Forward: CTC​ATT​CTG​TCT​CGA​GCC​CA
	Reverse: TGA​AGT​AGG​GAA​GGC​AGT​GG
TNF-α	Forward: GAG​CAC​GGA​AAG​CAT​GAT​CC
	Reverse: TAG​ACA​GAA​GAG​CGT​GGT​GG

IL, interleukin; TNF-α, tumor necrosis factor alpha.

### Detection of biochemical indicators

The supernatant of BALF was collected the protein levels in the lung tissue homogenate were determined using the BCA kit. Moreover, the activities of SOD and GSH-Px and the level of MDA in the lung tissue homogenate and the activity of LDH in BALF were measured based on the instructions of the kit.

### Untargeted metabolomics

A 100 mg sample of lung tissue was added to 500 μl of 80% methanol solution, vortexed, and shaken, and then left to stand in an ice bath for 5 min. Subsequently, the sample was centrifuged at 15,000 × *g* at 4°C for 20 min. The supernatant was collected and diluted with water to 53% methanol, followed by centrifugation at 15,000 g at 4°C for 20 min. The supernatant was collected to obtain a tissue homogenate, in which the level of metabolites was detected by liquid chromatography–mass spectrometry according to the specific detection method and analysis reported previously by the group (Xie et al., 2021, Supplementary materials).

### Statistical processing

The experimental results were analyzed using the SPSS Statistics 20.0 statistical software, and the measurement data were expressed as mean ± standard deviation. A *t*-test and a one-way analysis of variance with Tukey’s HSD (honest significant difference) post-hoc test were used for the comparison of means among multiple groups. Significance was set at *p* < 0.05.

## Results

### Identification of main compounds in LG by UPLC-MS analysis

Geniposide, menthol, isoorientin, baicalin, forsythin, forsythoside A, emodin and liquiritin were used as the reference standards to validate the main compounds in LG. The detailed information of these compounds were shown in Supplementary Figure S1. The typical based peak intensity (BPI) chromatograms of LG and these reference standards were shown in Supplementary Figure S2. The characteristic fragment ions of these compounds were shown in Supplementary Table S1. Emodin in *R. palmatum* L. [Polygonaceae; Rhei radix et rhizoma], liquiritin in *G. glabra* L. [Fabaceae; Glycyrrhizae radix et rhizoma], geniposide in *G. jasminoides* J. Ellis [Rubiaceae; Gardeniae fructus praeparatus], menthol in *M. canadensis* L. [Lamiaceae; Menthae haplocalycis herba], baicalin in *S. baicalensis* Georgi [Lamiaceae; Scutellariae radix], forsythin and forsythoside A in *F. suspensa* (Thunb.) Vahl [Oleaceae; Forsythiae fructus], and isoorientin in *L. gracile* Brongn [Poaceae; Lophatheri herba]. Were identified as the preeminent compounds in LG.

### Therapeutic effects of Liang-Ge decoction on sepsis-associated lung injury model rats

**FIGURE 2:**
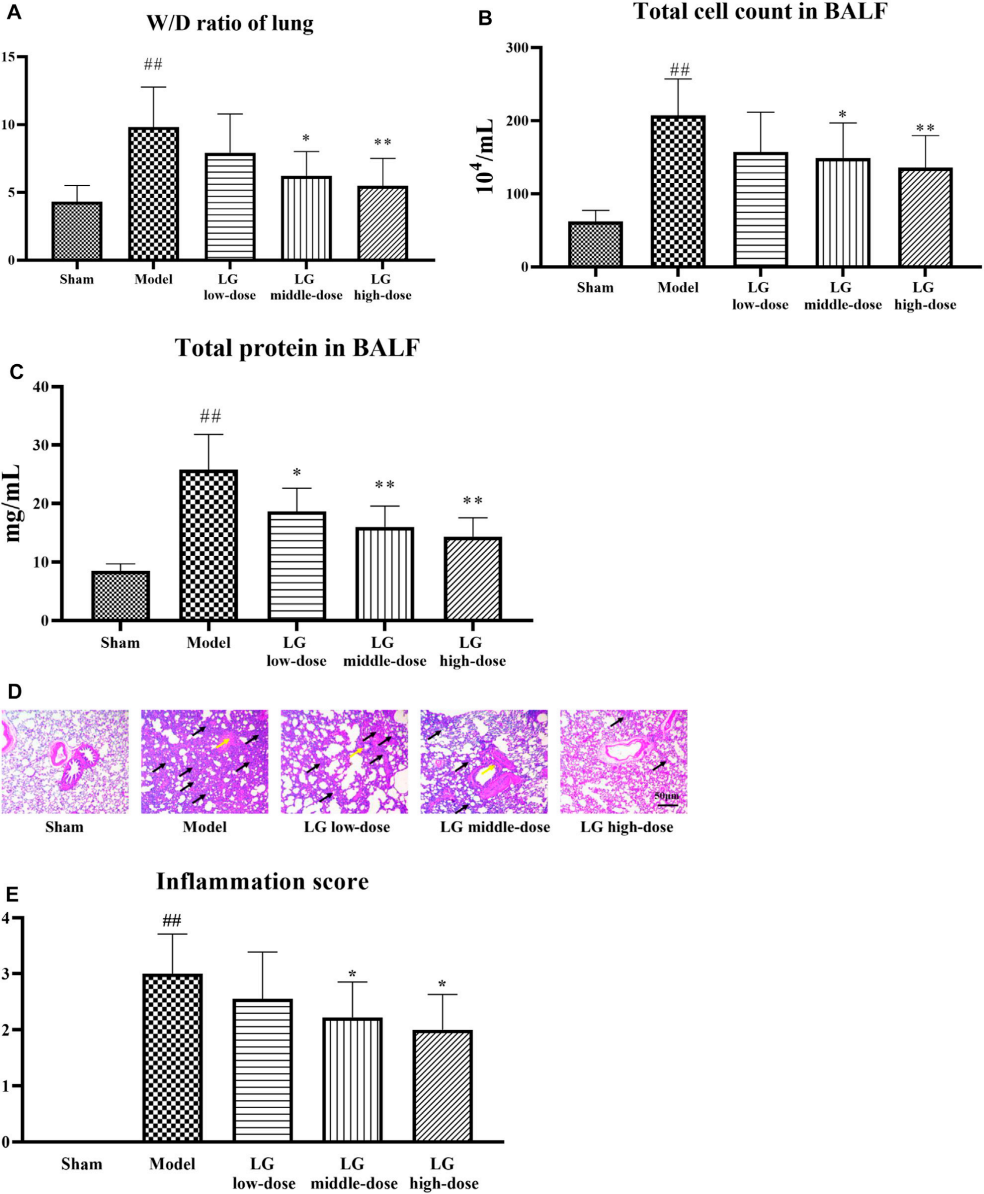
LG treatment alleviated acute lung injury in septic model rats. **(A)** LG decreased the W/D ratio in lung. **(B)** LG treatment decreased the total cell count in BALF. **(C)** LG treatment decreased the total protein concentration in BALF. **(D,E)** HE staining indicated that LG treatment alleviated the pathological changes and decreased the inflammation score in lung (100×), black arrows indicated in infiltratiion of inflammatory cells and yellow arrows indicated the damage of bronchial structure. W/D: wet/dry, HE: hematoxylin and eosin. Sham group (n = 15), Model group (n = 8), LG low-dose group (n = 9), LG middle-dose group (n = 9), LG high-dose group (n = 10) ##: *p* < 0.01 compared with the sham group; *: *p* < 0.05 compared with the model group; **: *p* < 0.01 compared with the model group.

After 24 h of drug administration for modeling, 15 rats in sham group, 8 rats in the model group, 9 rats in LG low-dose group, 9 rats in LG middle-dose group, 10 rats in LG high-dose group were survived. The W/D ratio of lung tissue, total cell count and total protein level in BALF were significantly increased in model group compared with the sham group (*p* < 0.01, respectively). Middle- and high-dose treatment of LG reduced the W/D ratio of lung tissue and total cell count in BALF in septic model rats (*p* < 0.05 and *p* < 0.01, respectively). In addition, the total protein levels in BALF were lower in LG low-dose (*p* < 0.05), LG middle-dose (*p* < 0.01) and LG high-dose (*p* < 0.01) groups compared with that in the model group (Figures 2A–C). Lung HE staining results showed intact bronchial epithelial structure, normal interalveolar septum, absence of interstitial edema in the lungs, and absence of significant inflammatory cell exudates in the sham group. In the model group, bronchial epithelial structure was no longer intact and infiltration by a considerable number of inflammatory cells could be observed. Treatment with low-, middle- and high-dose LG significantly improved pathological changes in lung from septic model rats (Figure 2D). Likewise, the inflammation score of HE staining was higher in model group than that in the control group and the inflammation score was lower in LG middle-dose (*p* < 0.05) and LG high-dose (*p* < 0.05) groups compared with that in the model group (Figure 2E).

### Effects of Liang-Ge decoction on inflammatory response, oxidative stress and apoptosis in lung in sepsis-associated lung injury model rats

**FIGURE 3:**
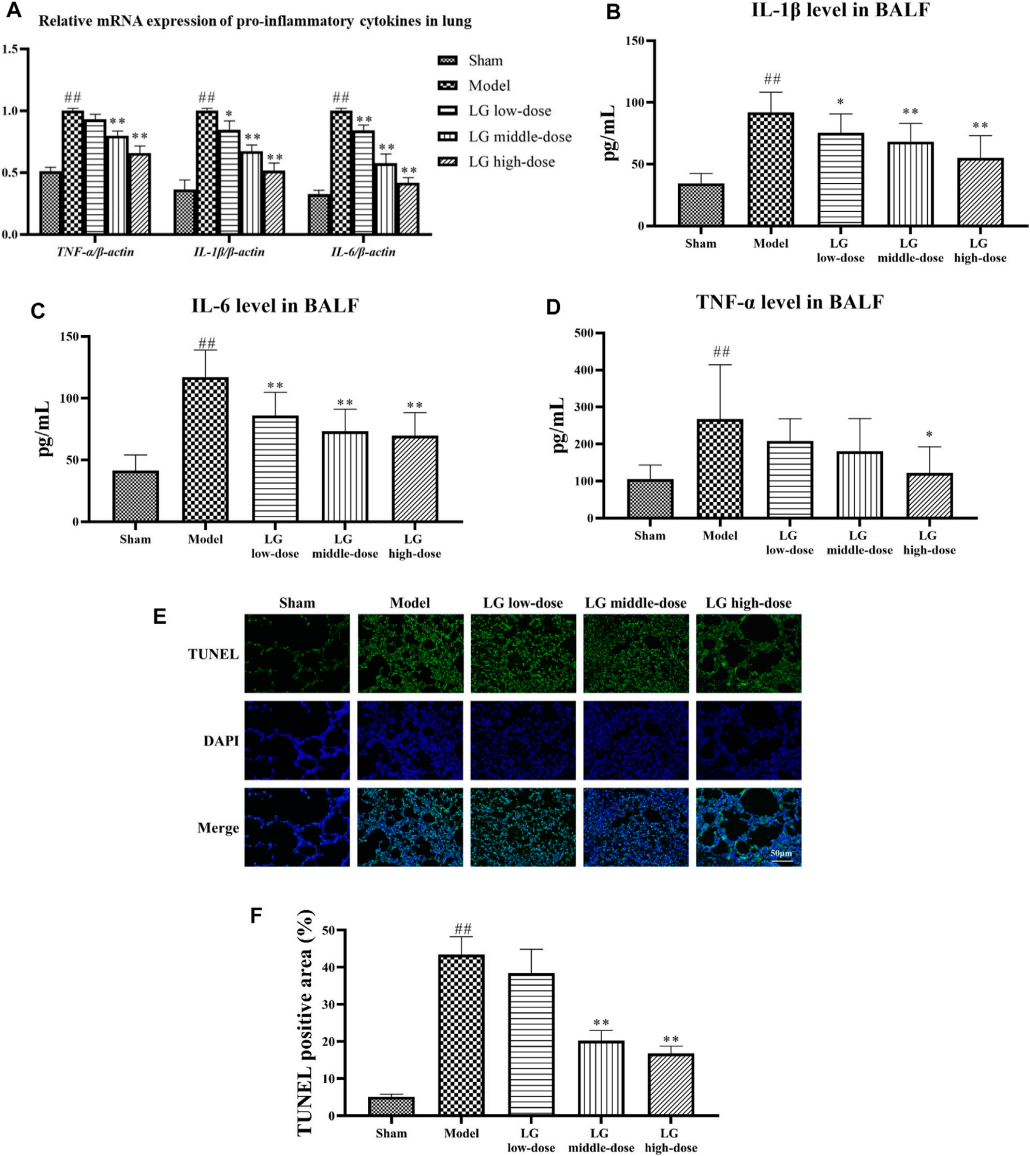
LG treatment reduced the inflammatory response and apoptosis in lung. **(A)** qPCR showed that LG treatment down-regulated the mRNA expression of *IL-6*, *IL-1β*, and *TNF-α* in lung. **(B–D)** LG treatment decreased the levels of IL-1β **(B)**, IL-6 **(C)** and TNF-α **(D)** in BALF. **(E,F)** TUNEL staining indicated that LG treatment decreased the proportions of apoptotic cells in lung (100×). TUNEL: terminal deoxynucleotidyl transferase deoxyuridine triphosphate (dUTP) nick end labeling. Sham group (n = 15), Model group (n = 8), LG low-dose group (n = 9), LG middle-dose group (n = 9), LG high-dose group (n = 10) ##: *p* < 0.01 compared with the sham group; *: *p* < 0.05 compared with the model group; **: *p* < 0.01 compared with the model group.

The anti-inflammatory effects of LG on sepsis-associated lung injury model rats were studied by investigating the gene and protein expressions of pro-inflammatory cytokines in lung and BALF. qPCR analysis showed that the mRNA expressions of *IL-6*, *IL-1β*, and *TNF-α* in lung tissue were up-regulated in model group compared with those in the sham group (*p* < 0.01, respectively). Compared with the model group, the mRNA expressions of *IL-1β* and *IL-6* were down-regulated in LG low-dose (*p* < 0.05 and *p* < 0.01, respectively), LG middle-dose (*p* < 0.01, respectively) and LG high-dose (*p* < 0.01, respectively) groups. The mRNA expression of *TNF-α* was lower in LG middle-dose and LG high-dose (*p* < 0.01, respectively) groups compared with the model group (Figure 3A). Likewise, ELISA results showed that the levels of IL-6, IL-1β, and TNF-α in BALF were increased in model group compared with those in the sham group (*p* < 0.01, respectively). Compared with the model group, the levels of IL-1β and IL-6 were lower in LG low-dose (*p* < 0.05 and *p* < 0.01, respectively), LG middle-dose (*p* < 0.01, respectively) and LG high-dose (*p* < 0.01, respectively) groups (Figures 3B,C). The level of TNF-α was decreased in LG high-dose groups compared with the model group (*p* < 0.05, Figure 3D).

Furthermore, the anti-oxidative effects of LG were evaluated by measuring the activities of SOD and GSH-Px and the level of MDA in lung tissue homogenates. The activities of SOD and GSH-Px were lower and the level of MDA was increased in model group compared with those in the sham group (*p* < 0.01, respectively). Compared with the model group, treatment with middle- and high-dose LG significantly elevated the activities of SOD (*p* < 0.05 and *p* < 0.01, respectively) and GSH-Px (*p* < 0.05 and *p* < 0.01, respectively) in lung tissue homogenates. The MDA level was lower in LG high-dose group compared with the model group (*p* < 0.05, Table 2).

**TABLE 2:** Acitivities of SOD and GSH-Px and level of MDA in lung tissue homogenates after LG treatment.

Group	SOD (U/mgprot)	MDA (nmol/mgprot)	GSH-Px (U/mgprot)
Sham	55.70 ± 14.92	1.73 ± 0.62	20.75 ± 3.80
Model	25.22 ± 15.6^##^	3.85 ± 1.35^##^	8.04 ± 4.58^##^
LG low-dose	34.56 ± 6.62	3.24 ± 1.35	12.22 ± 3.54
LG middle-dose	42.16 ± 13.07*	3.01 ± 1.03	12.37 ± 3.42*
LG high-dose	46.66 ± 13.62**	2.60 ± 0.62*	16.12 ± 3.88**

SOD, superoxide dismutase; MDA, methane dicarboxylic aldehyde; GSH-Px, glutathione peroxidase. Sham group (*n* = 15), Model group (*n* = 8), LG, low-dose group (*n* = 9), LG, middle-dose group (*n* = 9), LG, high-dose group (*n* = 10) ^##^ *p* < 0.01 compared with the sham group; *p < 0.05 compared with the model group; **p < 0.01 compared with the model group.

The anti-apoptotic effects of LG were then studied using TUNEL staining and by measuring the activity of LDH in BALF. TUNEL staining showed that the TUNEL positive area in model group were increased compared with the sham group (*p* < 0.01), whereas the TUNEL positive area in LG middle-dose and LG high-dose groups were decreased compared with the model group (*p* < 0.01, respectively, Figures 3E,F). Besides, the activity of LDH was increased in model group compared with the sham group (*p* < 0.01). Treatment with middle- and high-dose LG reduced the activity of LDH compared with the model group (*p* < 0.01, respectively, Table 3).

**TABLE 3:** Activity of LDH in BALF after LG treatment.

Group	LDH (U/mL)
Sham	4.37 ± 0.82
Model	31.27 ± 8.42^##^
LG low-dose	25.36 ± 4.24
LG middle-dose	21.25 ± 2.69**
LG high-dose	15.2 ± 1.61**

LDH, lactic dehydrogenase; Sham group (*n* = 15), Model group (*n* = 8), LG, low-dose group (*n* = 9), LG, middle-dose group (*n* = 9), LG, high-dose group (*n* = 10) ##: *p* < 0.01 compared with the sham group; **p < 0.01 compared with the model group.

Taken together, high-dose of LG treatment showed significant therapeutic, anti-inflammatory, anti-oxidative and anti-apoptotic effects on sepsis-associated lung injury model rats. Therefore, LG high-dose group was selected for further untargeted metabolomics study to elucidate the metabolic modulatory mechanisms of LG on sepsis-associated lung injury.

### Effects of Liang-Ge decoction on metabolite levels in lung in sepsis-associated lung injury model rats

**FIGURE 4:**
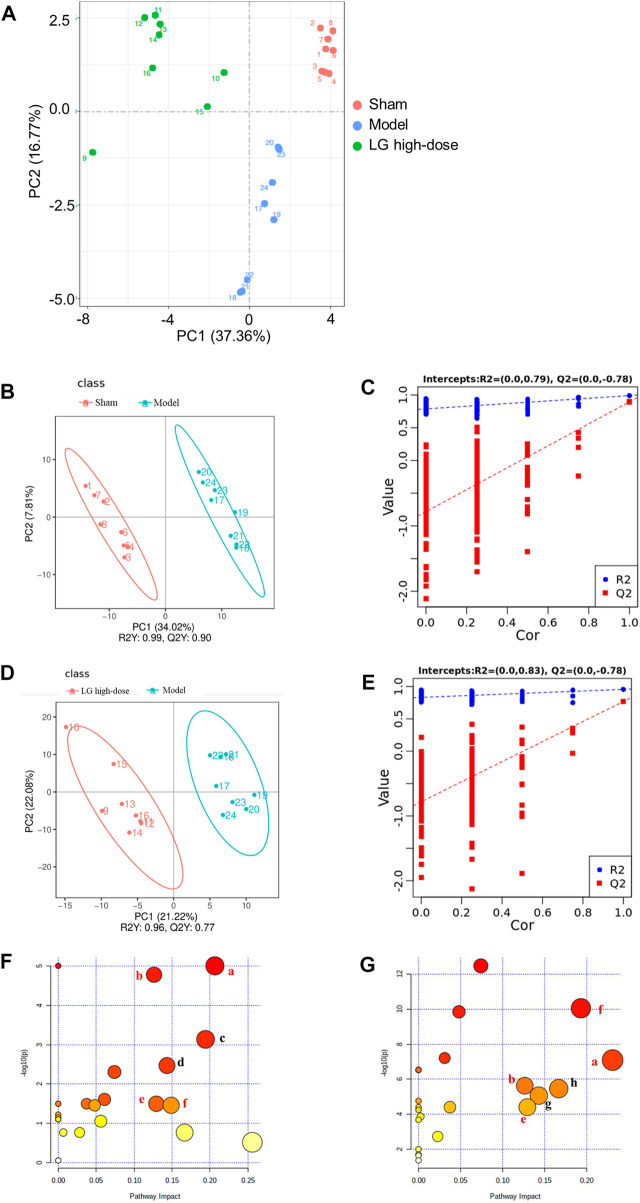
LG treatment modulated the metabolites in lung in sepsis-associated lung injury model rats. **(A)** Scores plots of PCA among each group. **(B,C)** Scores plots of PLS-DA between the sham and model groups and the corresponding coefficient of loading plots. **(D,E)** Scores plots of PLS-DA between the model and LG high-dose groups and the corresponding coefficient of loading plots. **(F,G)** Summary of pathway analysis between sham and model groups **(F)** and between model and LG high-dose groups **(G)**, the common pathways have been marked in red. **(A)** Glycine, serine and threonine metabolism; **(B)** Cysteine and methionine metabolism; **(C)** Nicotinate and nicotinamide metabolism; **(D)** Pyrimidine metabolism; **(E)** Inositol phosphate metabolism; **(F)** Citrate cycle (TCA cycle); **(G)** Tryptophan metabolism; **(H)** Aminoacyl-tRNA biosynthesis. Sham, model, and LG high-dose groups (n = 8 per group). PCA: principle component analysis, PLS-DA: partial least-squares discriminant analysis.

We further used untargeted metabolomics to study the changes of metabolites in lung sepsis-associated lung injury model rats after LG treatment. The principle component analysis (PCA) plot showed that the sham and model groups were well differentiated and that the model and LG high-dose group were also well differentiated (Figure 4A). To identify differentially expressed metabolites, partial least-squares discriminant analysis (PLS-DA) was used and the explanatory power (*R* ^2^) and predictive power (Q^2^) of PLS-DA model were accessed. Compared with the sham group, the model group had an *R* ^2^ = 0.79 and a Q^2^ = −0.78, whereas LG high-dose group had an *R* ^2^ = 0.83 and a Q^2^ = −0.78 compared with the model group (Figures 4B–E). These results showed that the model was stable and had good predictive power.

The following three criteria were used to screen for differentially expressed metabolites*: p* < 0.05 and VIP >1.0, fold change (FC) > 1.20 or FC < 0.80 (between sham and model group or between model and LG high-dose group), where 28 differentially expressed metabolites were identified in total (Table 4). Compared with those in the sham group, the levels of citric acid, glycocholic acid, hydroxyproline, cytidine, 2-deoxyuridine, uracil, cytosine, l-isoleucine, and DL-serine were significantly increased while those of cholesterol, 2-deoxycytidine, nicotinic acid, glutathione, oleanolic acid, nicotinamide, methionine, chenodeoxycholic acid, cholic acid, inositol, alpha-ketoglutaric acid and hippuric acid were significantly decreased in the model group. Levels of hippuric acid, alpha-ketoglutaric acid, inositol, cholic acid, chenodeoxycholic acid, methionine, oleanolic acid, nicotinic acid, l-ascorbate, threonine, D-proline and D-(+)-tryptophan were significantly increased in the LG high-dose group compared with those in the model group while those of DL-serine, DL-malic acid, taurodeoxycholic acid, l-isoleucine, inosine, cytosine, 2-deoxyuridine, cytidine, hydroxyproline, glycocholic acid and citric acid were significantly decreased in the LG high-dose group (Table 4).

**TABLE 4:** Differential metabolites in lung in sepsis-associated lung injury model rats after the treatment of LG.

No.	Formula	RT [min]	m/z	Metabolites	VIP		FC		Trend		Pathway
					M vs. S	L vs. M	M vs. S	L vs M	M vs. S	L vs. M	
1	C_6_H_8_O_7_	2.08	191.02	Citric acid	1.88	1.41	3.62	0.18	↑^##^	↓**	f
2	C_26_H_43_NO_6_	6.73	464.30	Glycocholic acid	1.97	1.02	4.77	0.36	↑^##^	↓**	
3	C_9_H_9_NO_3_	5.34	178.05	Hippuric acid	2.01	1.11	0.23	1.93	↓^##^	↑*	
4	C_5_H_6_O_5_	1.37	145.01	alpha-Ketoglutaric acid	1.63	1.62	0.55	4.84	↓^##^	↑**	f
5	C_6_H_12_O_6_	1.45	179.06	Inositol	1.43	1.55	0.68	5.61	↓^#^	↑**	e
6	C_24_H_40_O_5_	6.60	407.28	Cholic acid	1.38	1.16	0.28	2.97	↓^#^	↑**	
7	C_24_H_40_O_4_	6.91	437.29	Chenodeoxycholic Acid	1.18	0.52	0.39	1.89	↓^#^	↑*	
8	C_5_H_9_NO_3_	1.30	132.07	Hydroxyproline	1.86	0.62	3.91	0.15	↑^##^	↓**	
9	C_5_H_11_NO_2_S	5.30	150.06	Methionine	1.02	2.00	0.57	2.27	↓^##^	↑**	b, h
10	C_6_H_6_N_2_O	1.89	123.06	Nicotinamide	1.52	0.46	0.27	1.53	↓^##^	↑	c
11	C_30_H_48_O_3_	9.46	439.35	Oleanolic acid	1.51	1.02	0.57	3.19	↓^##^	↑**	
12	C_9_H_13_N_3_O_5_	1.38	244.09	Cytidine	1.21	0.78	2.17	0.28	↑^##^	↓**	d
13	C_10_H_17_N_3_O_6_S	1.38	308.09	Glutathione	1.32	0.48	0.58	1.52	↓^#^	↑	
14	C_9_H_12_N_2_O_5_	6.21	229.08	2-Deoxyuridine	1.42	0.29	3.09	0.23	↑^#^	↓*	d
15	C_4_H_4_N_2_O_2_	1.79	113.03	Uracil	1.30	0.86	1.99	0.55	↑^#^	↓	d
16	C_4_H_5_N_3_O	1.38	112.05	Cytosine	1.13	0.35	1.98	0.47	↑^##^	↓**	
17	C_6_H_5_NO_2_	1.74	124.04	Nicotinic Acid	1.21	1.75	0.46	2.49	↓^#^	↑*	c
18	C_9_H_13_N_3_O_4_	1.82	228.10	2-Deoxycytidine	1.07	0.38	0.53	1.82	↓^#^	↑	d
19	C_27_H_46_O	8.18	387.36	Cholesterol	1.22	0.39	0.56	1.68	↓^##^	↑	
20	C_6_H_8_O_6_	5.50	177.04	l-Ascorbate	0.18	1.64	0.96	1.65	↓	↑**	
21	C_10_H_12_N_4_O_5_	5.04	269.09	Inosine	0.22	1.80	1.16	0.44	↑	↓**	
22	C_6_H_13_NO_2_	3.01	132.10	l-Isoleucine	1.04	1.43	1.31	0.64	↑^#^	↓**	h
23	C_4_H_9_NO_3_	1.31	120.07	Threonine	0.62	1.38	0.79	4.20	↓	↑**	a, h
24	C_5_H_9_NO_2_	1.37	116.07	D-Proline	0.71	1.14	0.70	1.79	↓	↑**	
25	C_26_H_45_NO_6_S	7.13	500.30	Taurodeoxycholic Acid	0.24	1.44	1.35	0.35	↑	↓**	
26	C_4_H_6_O_5_	1.67	133.01	DL-Malic acid	0.61	1.30	1.21	0.35	↑	↓**	f
27	C_11_H_12_N_2_O_2_	5.13	157.08	D-(+)-Tryptophan	0.62	1.43	0.85	2.99	↓	↑**	g, h
28	C_3_H_7_NO_3_	1.29	106.05	DL-Serine	1.05	1.59	2.11	0.23	↑^##^	↓**	a, b, h

RT, retention time; VIP, variable importance of projection; FC: fold change; ^#^ *p* < 0.05 as compared to the control group; ^##^ *p* < 0.01 as compared to the control group; *p < 0.05 as compared to the model group; ^**^ *p* < 0.01 as compared to the model group; ↑content increased; ↓content decreased; vs. S, sham group; M, model group; L: LG, high-dose group. a, Glycine, serine and threonine metabolism; b, Cysteine and methionine metabolism; c, Nicotinate and nicotinamide metabolism; d, Pyrimidine metabolism; e, Inositol phosphate metabolism; f, Citrate cycle (TCA, cycle); g, Tryptophan metabolism; h, Aminoacyl-tRNA, biosynthesis. Sham, model, and LG, high-dose groups (*n* = 8 per group)^. #^ *p* < 0.05 compared with the sham group; ^##^ *p* < 0.01 compared with the sham group; *p < 0.05 compared with the model group; **p < 0.01 compared with the model group.

### Pathway analysis of differential metabolites

The MetaboAnalyst platform was used for metabolic pathway enrichment analysis of differentially expressed metabolites together with the KEGG database. Differential metabolic pathways were selected based on a pathway impact >0.05 and *p* < 0.05 (Xie et al., 2021). Differential metabolic pathways between the sham group and model group included glycine, serine and threonine metabolism, cysteine and methionine metabolism, nicotinate and nicotinamide metabolism, pyrimidine metabolism, inositol phosphate metabolism, citrate cycle (TCA cycle) (Figure 4F). Differential metabolic pathways between the model group and the LG high-dose group included glycine, serine and threonine metabolism, cysteine and methionine metabolism, inositol phosphate metabolism, TCA cycle, tryptophan metabolism and aminoacyl-tRNA biosynthesis (Figure 4G). Among these pathways, glycine, serine and threonine metabolism, cysteine and methionine metabolism, inositol phosphate metabolism and TCA cycle were pathways common to the sham, model, and LG high-dose groups.

## Discussion

In this study, we used CLP to generate a rat model of sepsis. Our results showed that the W/D ratio of the lung tissues in the model group rats was increased, along with the increase of total cell count and protein levels in the BALF, suggesting increased permeability of the lung tissues in the model group rats. The results of the pathological staining revealed a significant inflammatory cell infiltration in the lung tissue of the model group rats, which was accompanied by bronchial epithelial cell injury. These changes were consistent with the pathological changes of sepsis-associated lung injury (Jia et al., 2022). LG significantly reduced the permeability of the lung tissues in rats with sepsis-associated lung injury and improved the pathological changes detected in the lung tissues.

Our results also showed that LG downregulated the expression of the pro-inflammatory cytokines IL-6, IL-1β, and TNF-α in lung tissues, and decreased the levels of these cytokines in the BALF. The overactivation of inflammatory responses is an important pathological process in the early stages of sepsis, and these responses induced by infection and injury are initially protective, but can severely damage lung tissues when the stimulus is too intense and cytokine production is excessive (Jianjun et al., 2018). Studies have shown that the levels of pro-inflammatory cytokines, such as those of IL-6, IL-1β, and TNF-α, significantly increase in rats from 6 to 48 h after CLP treatment (Zafrani et al., 2012; Jia et al., 2021).

In addition, oxidative stress is among the pathological manifestations of lung injury in sepsis. Bacterial infection can directly cause excessive reactive oxygen species (ROS) production in lung tissues, and the high levels of inflammatory cytokines can also promote ROS production; in turn, these ROS can damage membrane structures in lung tissue cells through oxidative reactions (Tripathi et al., 2018). Our results showed that LG increased the activity of SOD and GSH-Px and decreased MDA levels in lung tissues. MDA is a lipid peroxidation end product that is cytotoxic and its level is positively correlated with the degree of oxidative stress (Tsikas, 2017). SOD and GSH-Px are important antioxidant enzymes that scavenge ROS from cells (Olsvik et al., 2005); SOD promotes the conversion of ROS to H_2_O_2_, which is subsequently converted to H_2_O and O_2_ under the effect of GSH-Px. The increased activity of SOD and GSH-Px protects the lung tissues from oxidative stress injury (Liu et al., 2022).

Bacterial infection can directly lead to alveolar epithelial cell necrosis and apoptosis, and the excessive inflammatory responses and oxidative stress induced by infection can also aggravate alveolar cell damage, which in turn can impair the barrier function of lungs and alter the permeability of lung tissues. LG reduces the activity of LDH in the BALF. LDH is an important regulatory enzyme of anaerobic glycolysis and gluconeogenesis. The disruption of the cell membrane structure causes LDH release; thus, the detection of LDH can reflect cell injury (Laganá et al., 2019). In addition, we used TUNEL staining to observe apoptosis in lung tissues. After apoptosis, the chromosomes break, producing a large number of sticky 3′-OH ends. TUNEL staining is a method that is commonly used to detect apoptotic cells via terminal deoxynucleotidyl transferase to label the sticky 3′-OH ends of DNA with derivatives formed from dUTP and fluorescein (Mirzayans and Murray, 2020). The results of TUNEL staining showed a decrease in the number of apoptotic cells in lung tissues after the LG intervention.

Next, we investigated the effect of LG on metabolites in the lung tissues of rats with sepsis-associated lung injury using untargeted metabolomics. A PCA and PLS-DA revealed significant changes in lung tissue metabolism in the model and sham groups, with the lung tissue metabolism in rats with sepsis-associated lung injury being significantly affected after treatment with LG. A further differential metabolite analysis showed that LG affected the levels of 23 metabolites in lung such as citric acid, methionine, threonine, alpha-ketoglutaric acid, and inositol. A metabolic pathway analysis of the differential metabolites using MetaboAnalyst showed that glycine, serine and threonine metabolism, cysteine and methionine metabolism, inositol phosphate metabolism and TCA cycle pathways were the common pathways showing differences between the sham and model groups and between the model and LG high-dose groups, suggesting that LG plays a role in the treatment of sepsis-associated lung injury by regulating the metabolites in these pathways.

### Glycine, serine, and threonine metabolism

Our results showed that the LG intervention reduced DL-serine levels and increased threonine levels in the glycine, serine, and threonine metabolism pathway in the lung tissues of rats with sepsis-associated lung injury. Amino acid metabolism is closely related to immune cell activation in inflammatory responses. Serine belongs to the one-carbon unit amino acids and plays an important role in cellular nucleotide synthesis (Yang et al., 2019; Yang and Vousden, 2016). In a lipopolysaccharide-induced macrophage model, serine supplementation promoted IL-1β secretion, whereas the inhibition of serine metabolism reduced IL-1β production in a sepsis model, while improving survival in septic mice (Rodriguez et al., 2019). The inhibition of IL-1β production by LG in rats with sepsis-associated lung injury may be associated with a decrease of serine levels in macrophages; however, this needs to be confirmed in additional *in vitro* studies. Threonine is an essential amino acid and an important nutrient for the body and can be converted to acetyl-CoA to enter the TCA cycle and provide energy to the body (Tang et al., 2021). The role of threonine in sepsis has not been reported, and the relationship between the increased threonine levels after treatment with LG and improved sepsis-associated lung injury warrants further study.

### Cysteine and methionine metabolism

Our results showed that the LG intervention reduced DL-serine levels and increased methionine levels in the cysteine and methionine metabolism pathway in the lung tissues of rats with sepsis-associated lung injury. The relationship between serine and sepsis-associated lung injury has been discussed previously in this study. Methionine can be converted from serine, which plays an important regulatory role in cell survival (Lee et al., 2015). An *in vitro* study found that methionine deficiency caused apoptosis and cell-cycle arrest (Song et al., 2021). LG may inhibit the inflammatory responses and apoptosis of lung tissue by promoting the conversion of serine to methionine.

### Inositol phosphate metabolism

Our results showed that the LG intervention increased inositol levels in the inositol phosphate metabolism pathway in the lung tissues of rats with sepsis-associated lung injury. Inositol is an important nutrient that promotes cell survival and proliferation and is also used as a drug in the alleviation of respiratory distress syndrome (RDS), Alzheimer’s disease, etc. (Bizzarri et al., 2016). An ensuing shamled clinical study found that supplementation with inositol in preterm infants presenting with RDS significantly improved clinical symptoms and reduced the incidence of sepsis in the affected children (Howlett et al., 2019). In addition, an *in vitro* study found that Myo-inositol, which is an inositol derivative, protects cells from oxidative stress injury (Ponchia et al., 2021). Similarly, one study found that *in vitro* intervention using Myo-inositol combined with ethanolamine significantly alleviated H_2_O_2_-induced oxidative stress injury in cells (Sibomana et al., 2019). Therefore, the effect of LG in ameliorating oxidative stress in sepsis-associated lung injury may be related to the increase in inositol levels in the inositol phosphate metabolism pathway.

### TCA cycle

Our results showed that LG increased the level of alpha-ketoglutaric acid and decreased those of citric acid and DL-malic acid in the TCA cycle in the lung tissues of rats with sepsis-associated lung injury. The TCA cycle is the most efficient way for the body to oxidize carbohydrates for energy and is the hub for the metabolic liaison and transformation of carbohydrates, lipids, and amino acids. A recent study showed that the reprogramming of glucose metabolism is closely related to macrophage polarization (Russo et al., 2021). Moreover, an *in vitro* study revealed that alpha-ketoglutaric acid inhibits M1-type macrophage polarization and reduces the production of the pro-inflammatory cytokines IL-1β, IL-6, and TNF-α by inhibiting nuclear factor kappa B (NF-κB) pathway activation, and also promotes M2-type macrophage polarization by activating Jumonji domain-containing protein D3 (JMJD3) (Pu-Ste et al., 2017). In contrast, citric acid and DL-malic acid levels were significantly increased in activated macrophages (Viola et al., 2019). Citric acid promotes macrophage activation, which in turn induces inflammatory responses (Viola et al., 2019). Interestingly, a recent study found that the citrate/malate exchange on macrophage mitochondria plays an important role in regulating macrophage activation and the secretion of inflammatory mediators (Palmieri et al., 2015). The reprogramming of carbohydrate metabolism in macrophages through its regulation provides a novel strategy for inhibiting the development of inflammatory responses. Melatonin can inhibit the development of inflammatory responses by increasing the levels of alpha-ketoglutaric acid in macrophages (Liu et al., 2017).

In conclusion, our study demonstrated the therapeutic effetcts of LG on sepsis-associated lung injury model rats. Moreover, LG could inhibit the inflammatory response, oxidative stress and apoptosis and regulate metabolites related to glycine, serine and threonine metabolism, cysteine and methionine metabolism, inositol phosphate metabolism and TCA cycle in lung in sepsis-associated lung injury model rats. This study is the first to screen the differential metabolites of LG on CLP-induced sepsis-associated lung injury model. In addition, our study revealed significant anti-inflammatory, anti-oxidative and anti-apoptotic potentials of LG. Based on the close relationship between host metabolism and pathological processes such as inflammatory response, oxidative stress and apoptosis, our further studies should be carried out using *in vivo* and *in vitro* model to construct a metabolism-downstream pathway regulatory network of LG and its active components on sepsis-associated lung injury model.

## Data Availability

The original contributions presented in the study are included in the article/Supplementary Material, further inquiries can be directed to the corresponding author.
