# Supplementary material for: Liang-Ge decoction ameliorates acute lung injury in septic model rats through reducing inflammatory response, oxidative stress, apoptosis, and modulating host metabolism
Source: Front Pharmacol. 2022 Sep 16;13:926134. doi: 10.3389/fphar.2022.926134 (PMC9523795; doi:10.3389/fphar.2022.926134)
Supplement: Supplementary file 1 [file DataSheet1.docx]

**Supplementary materials**

**Methods**

**Experimental conditions of untargeted metabolomics analysis**

**Conditions of chromatography and mass spectrometry**

Chromatography was performed using a Hypesil Goldcolumn (C18) chromatographic column (2.1 mm × 100 mm, 1.9 μm) with a mobile phase consisting of (A) 0.1% formic acid and (B) methanol, using a gradient elution of 0 min, 98% A, 2% B; 1.5 min, 98 % A, 2% B; 12 min, 0% A, 100% B; 14 min, 0% A, 100% B; 14.1 min, 98% A, 2% B; and 17 min, 98% A, 2% B. The column temperature was set at 40°C, flow rate was 0.2 mL/min, and injection volume was 2 μL.

Mass spectrometry conditions involved simultaneous detection using the positive and negative ion modes of electrospray ionization (ESI). The ESI source settings were as follows: spray voltage, 3.2 kV; sheath gas flow rate, 40 arb; auxiliary gas flow rate, 10 arb; capillary temperature, 320°C; polarity, positive or negative; selection of scan range, 100–1500 m/z; MS/MS secondary scan was data-dependent scans. All the samples were obtained in aliquots and mixed and this was used as the quality control (QC) sample. Periodic analysis was performed to monitor the instrument’s stability throughout the analysis process. Throughout the experiment, QC was added after running every six samples to assess the stability of the experiment.

**Data processing and analysis**

The detection of characteristic molecular peaks in the samples was based on the high-resolution mass spectrometry detection technique. The molecular peaks were matched and identified using a combination of high-quality mzCloud, mzVault, and MassList databases constructed from the standards. The raw files (.raw) obtained using mass spectrometry were imported into Compound Discoverer 3.1 (CD3.1, Thermo Fisher) software for data preprocessing. First, the data were preliminarily screened by retention time, mass-to-charge ratio, and other parameters. Then the peaks were aligned based on the retention time deviation of 0.2 min and mass deviation of 5 parts per million (ppm) for different samples to improve identification accuracy. Next, peak extraction was performed based on the setting information of 5 ppm, signal intensity deviation of 30%, signal-to-noise ratio of 3, and minimum signal intensity of 100,000. The adduct ions and peak area were simultaneously quantified. The metabolites were then identified through molecular formula prediction using molecular ion peaks and fragment ions and compared with the mzCloud, mzVault, and MassList databases. Metabolites with a coefficient of variance of less than 30% in the QC samples were retained as final identification for subsequent analyses. The peaks detected in these samples were integrated using CD3.1 software where the peak area of each characteristic peak represented the relative quantitative value of a metabolite. The quantitative results were normalized using the total peak area, and finally, the quantitative results of the metabolites were obtained. The data were then subjected to QC to ensure the accuracy and reliability of the data results. Next, multivariate statistical analysis of the metabolites was performed, including principal component analysis (PCA) and partial least squares discriminant analysis (PLS–DA), to discover any differences in metabolic patterns among the different groups. Metabolite correlation analysis revealed the relationship within the samples and metabolites. Finally, the biological significance of the metabolite correlation was explained by functional analysis such as metabolic pathway analysis. The present study screened potential biomarkers based on *P* ≤0.05 and variable importance of projection (VIP) >1, fold change (FC) >1.20 or FC < 0.80 (between sham and model group or between model and LG high-dose group). Metabolic pathway enrichment analysis was performed for differential metabolites based on KEGG database.

mzCloud: https://www.mzcloud.org/

MetaboAnalyst: https://www.metaboanalyst.ca/

Kyoto Encyclopedia of Genes and Genomes (KEGG): <https://www.kegg.jp/>

**Results**


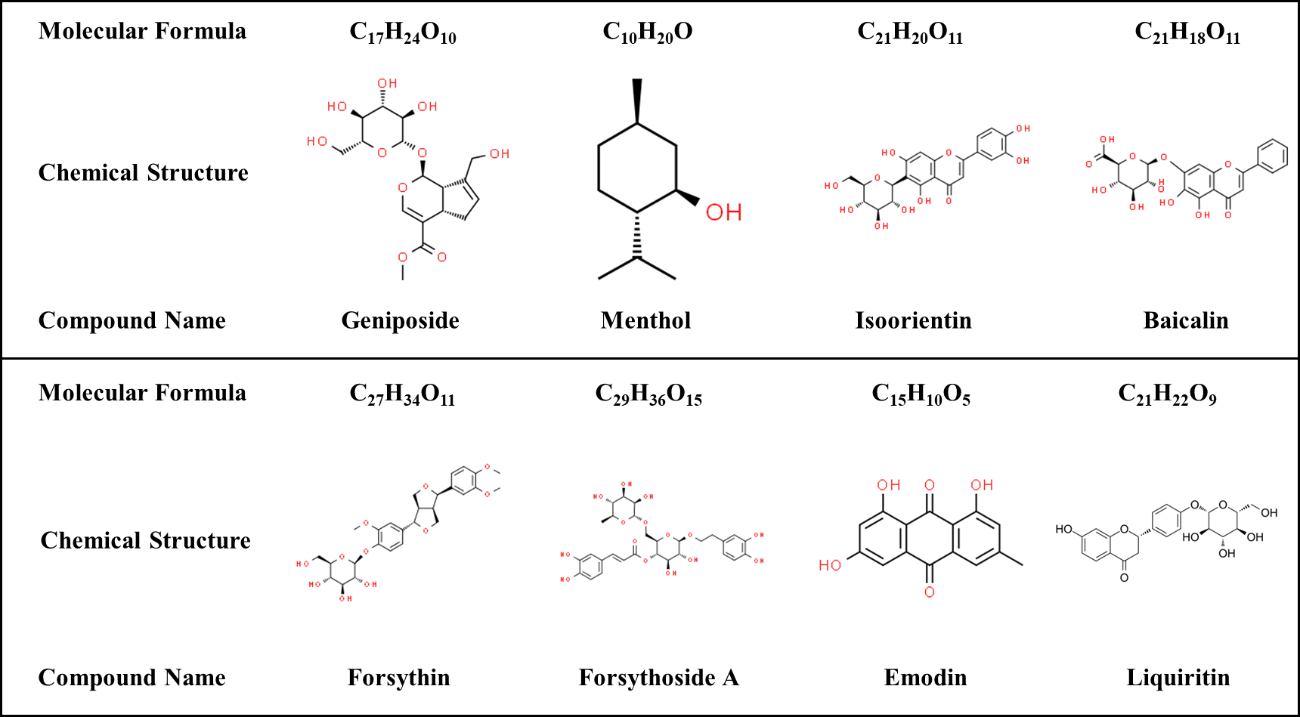


**FIGURE S1:** The molecular formulas and chemical structures of reference standards.

a


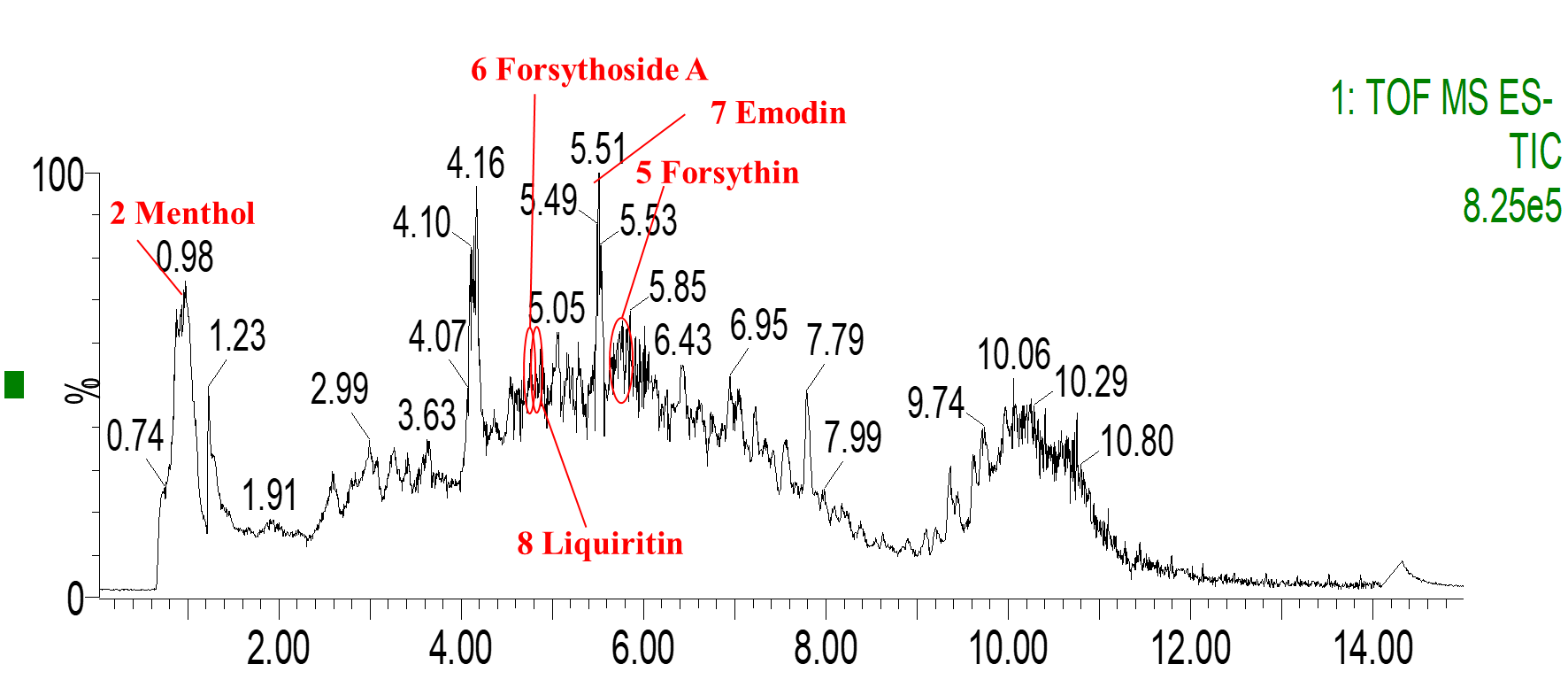


b


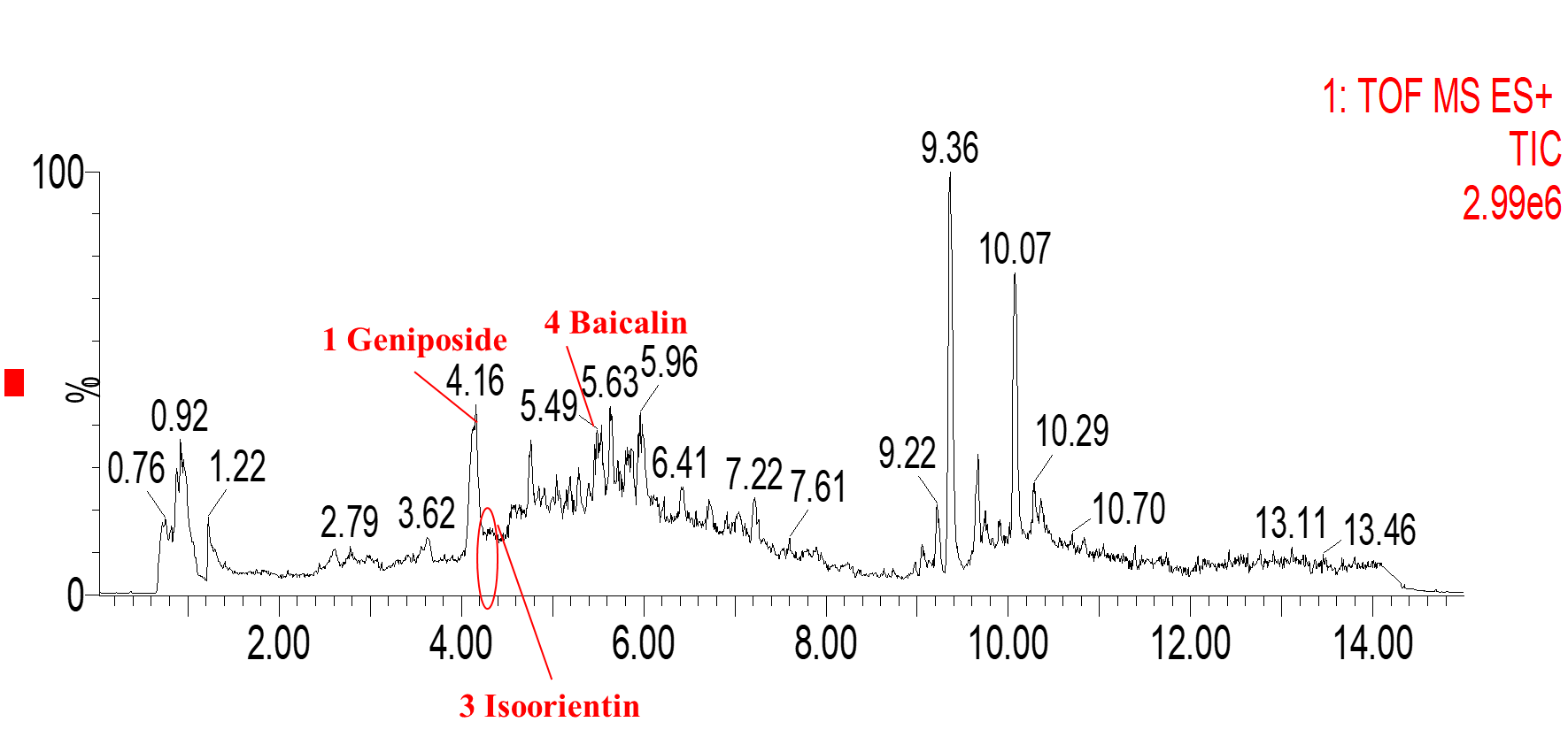


c


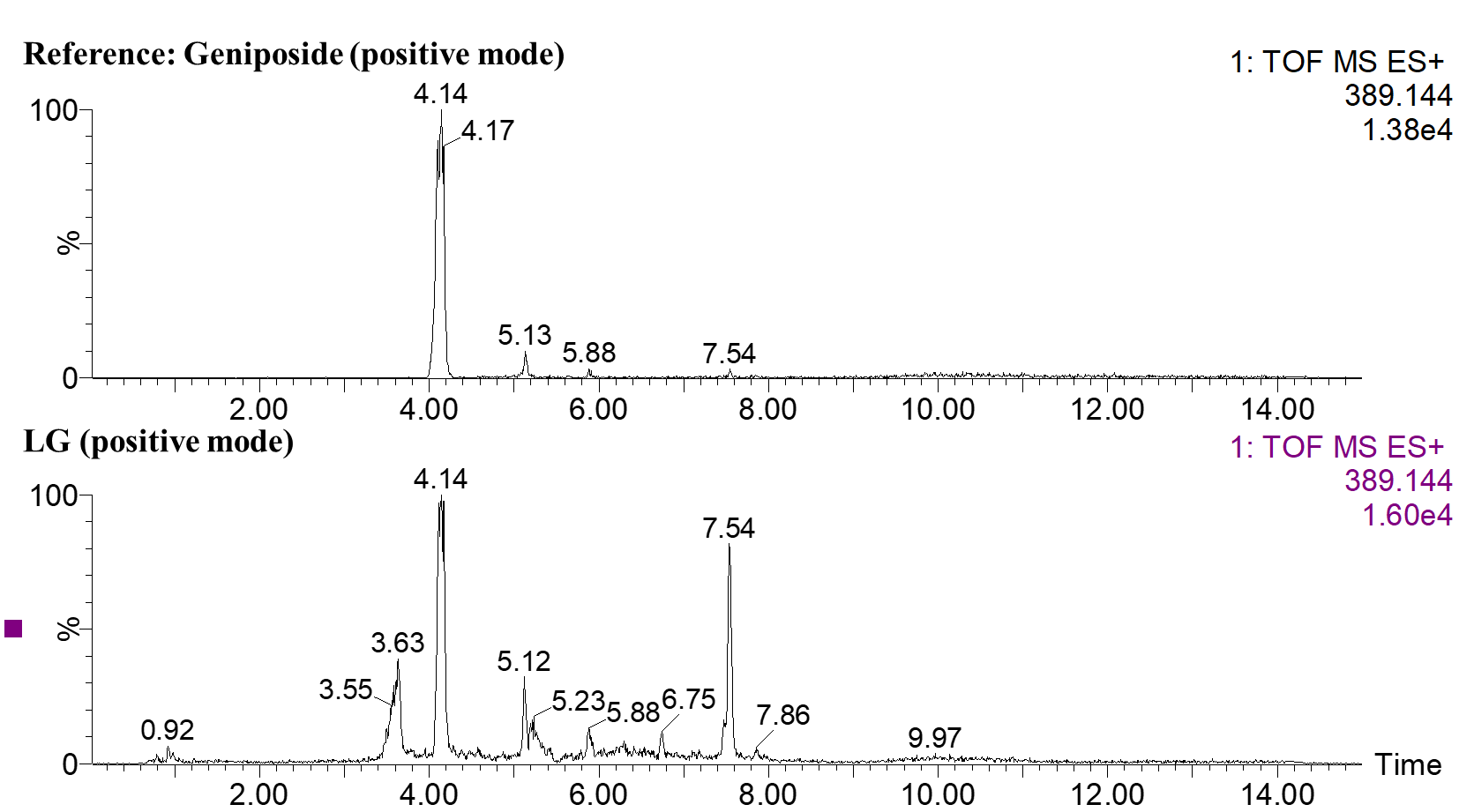


d


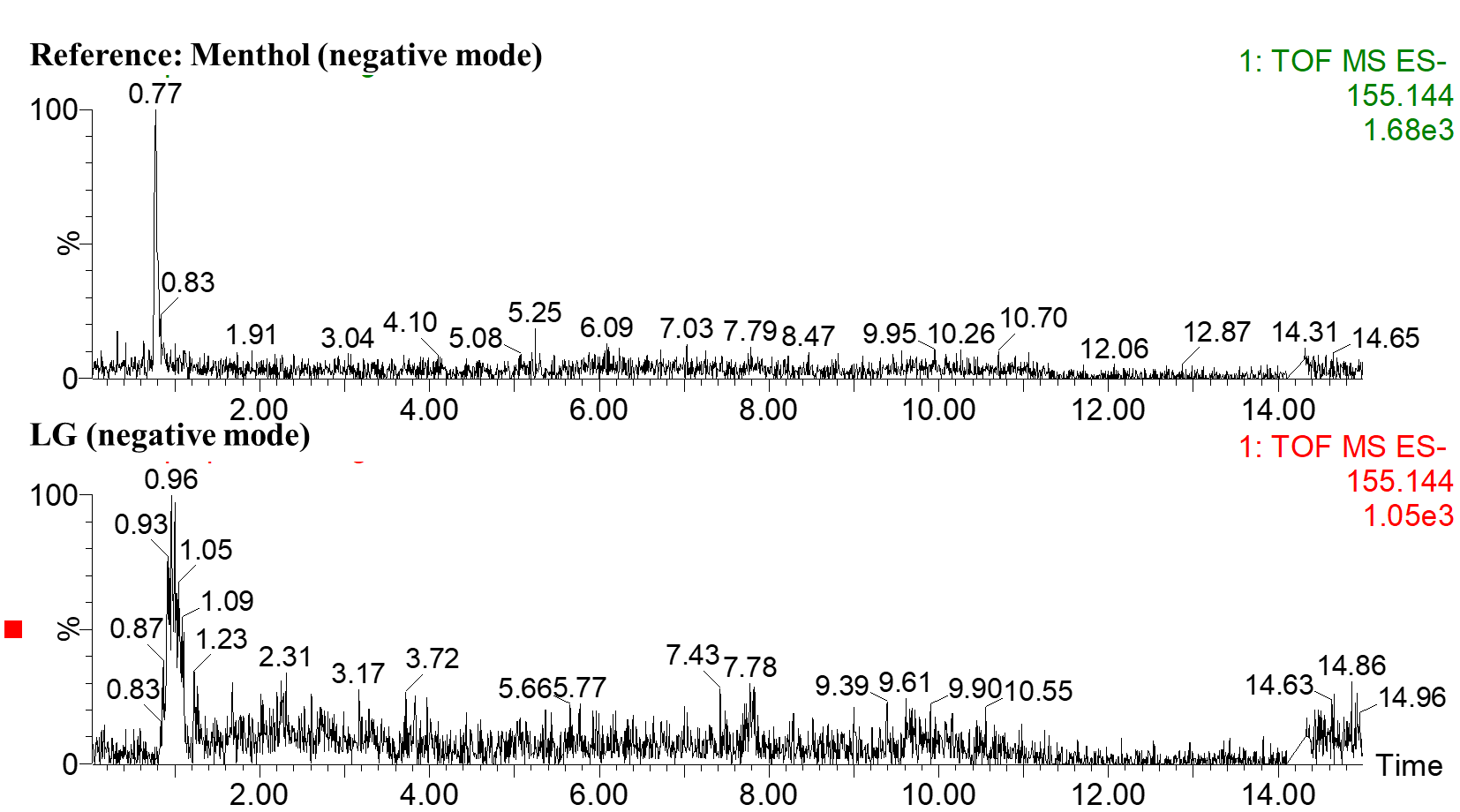


e


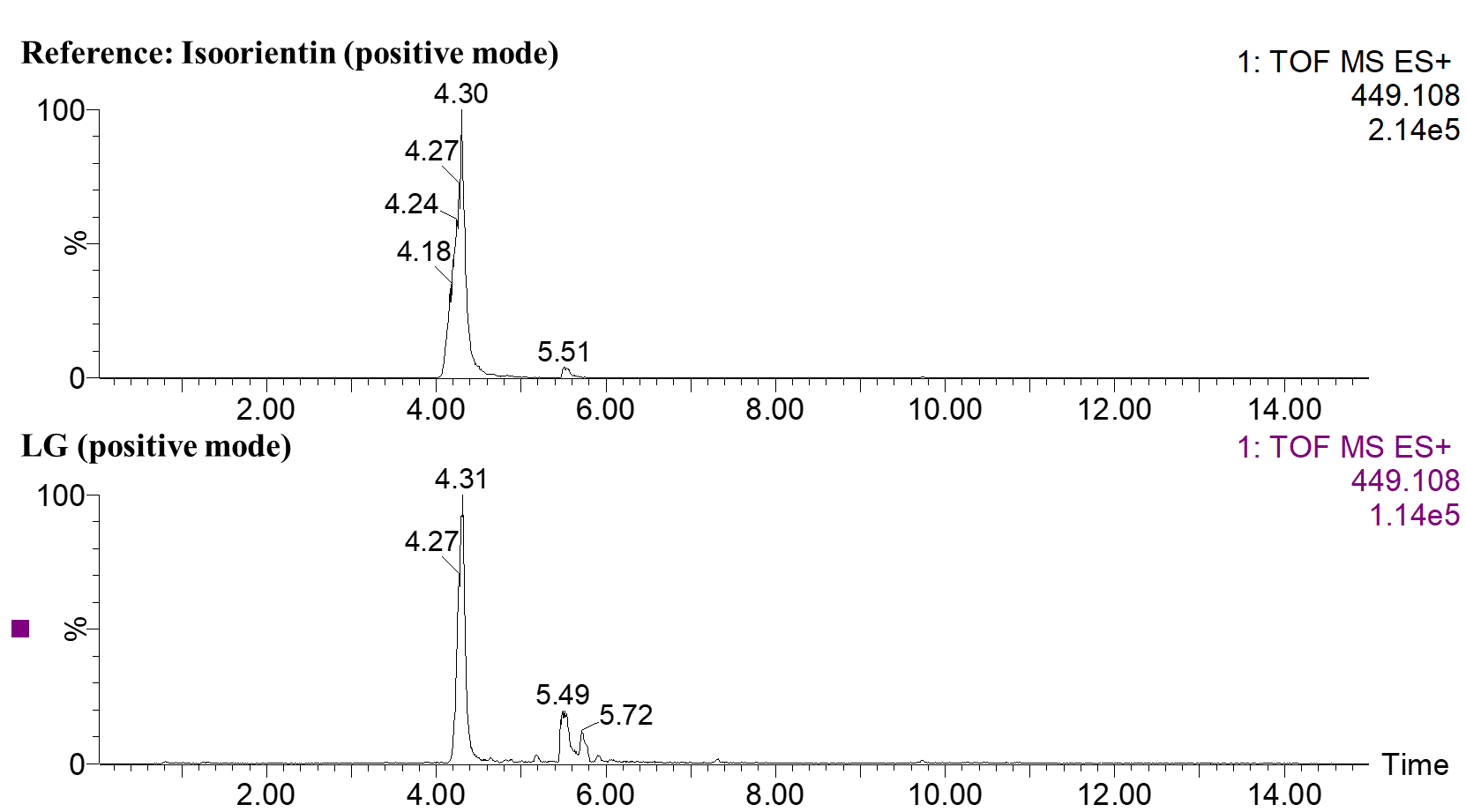


f


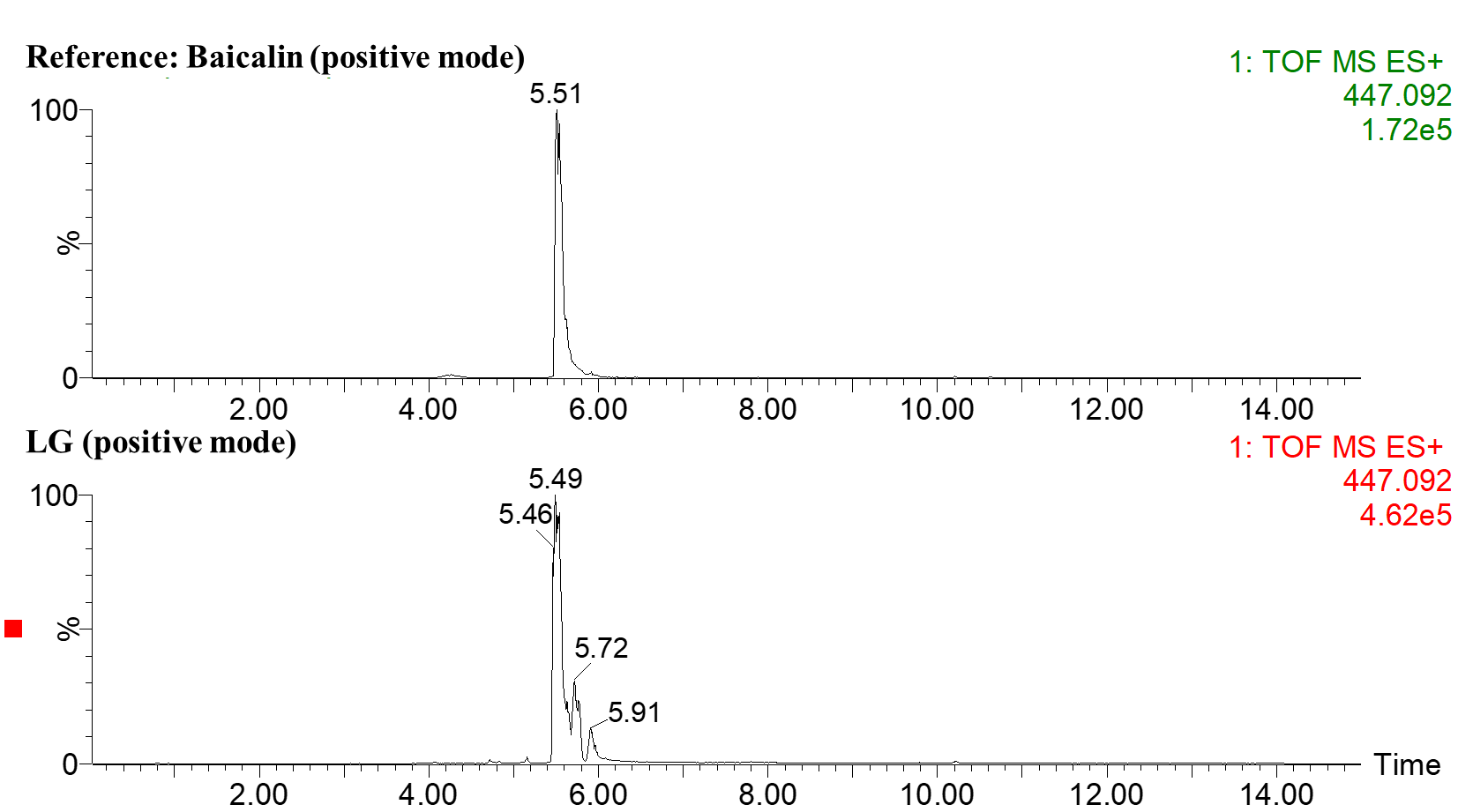


g


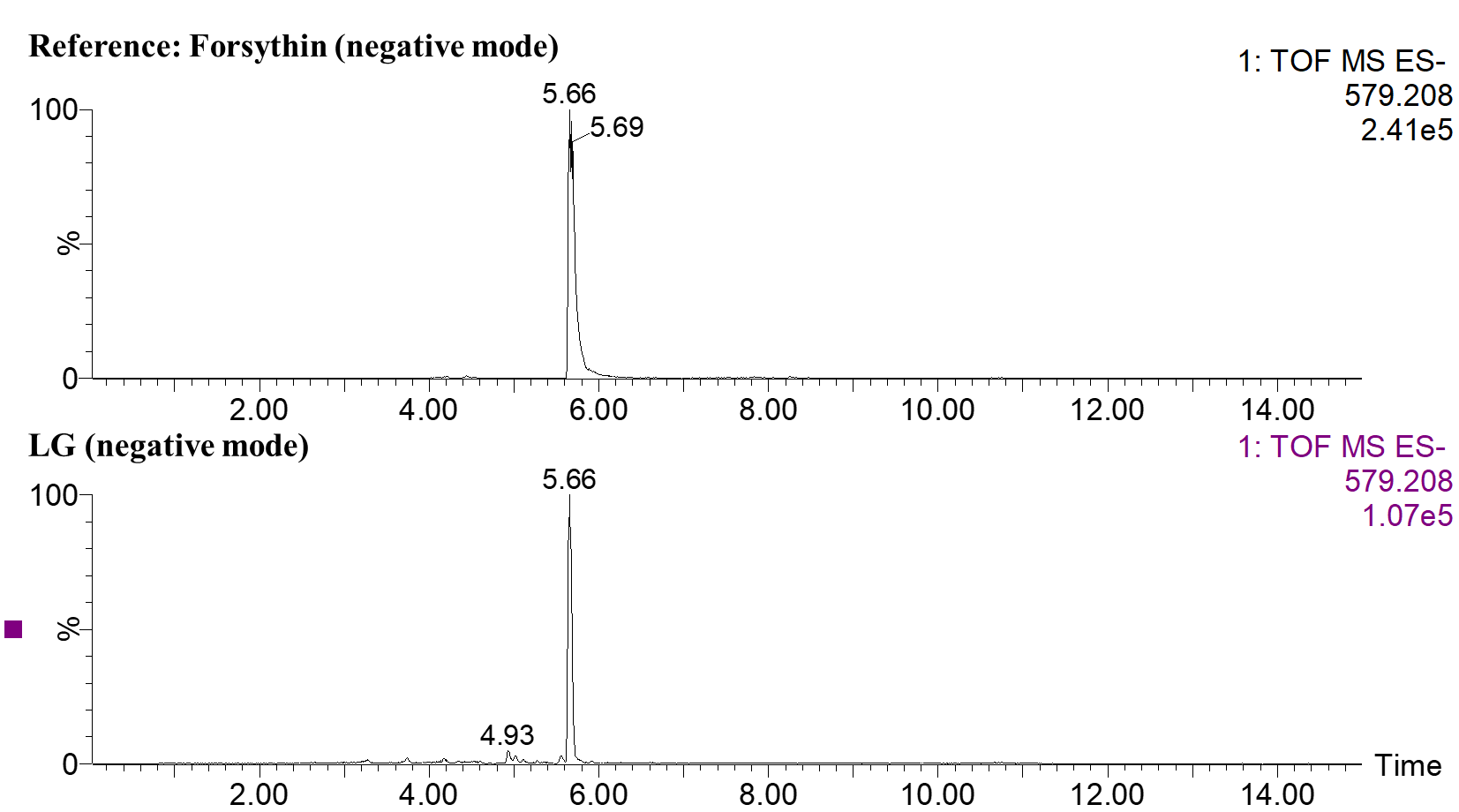


h


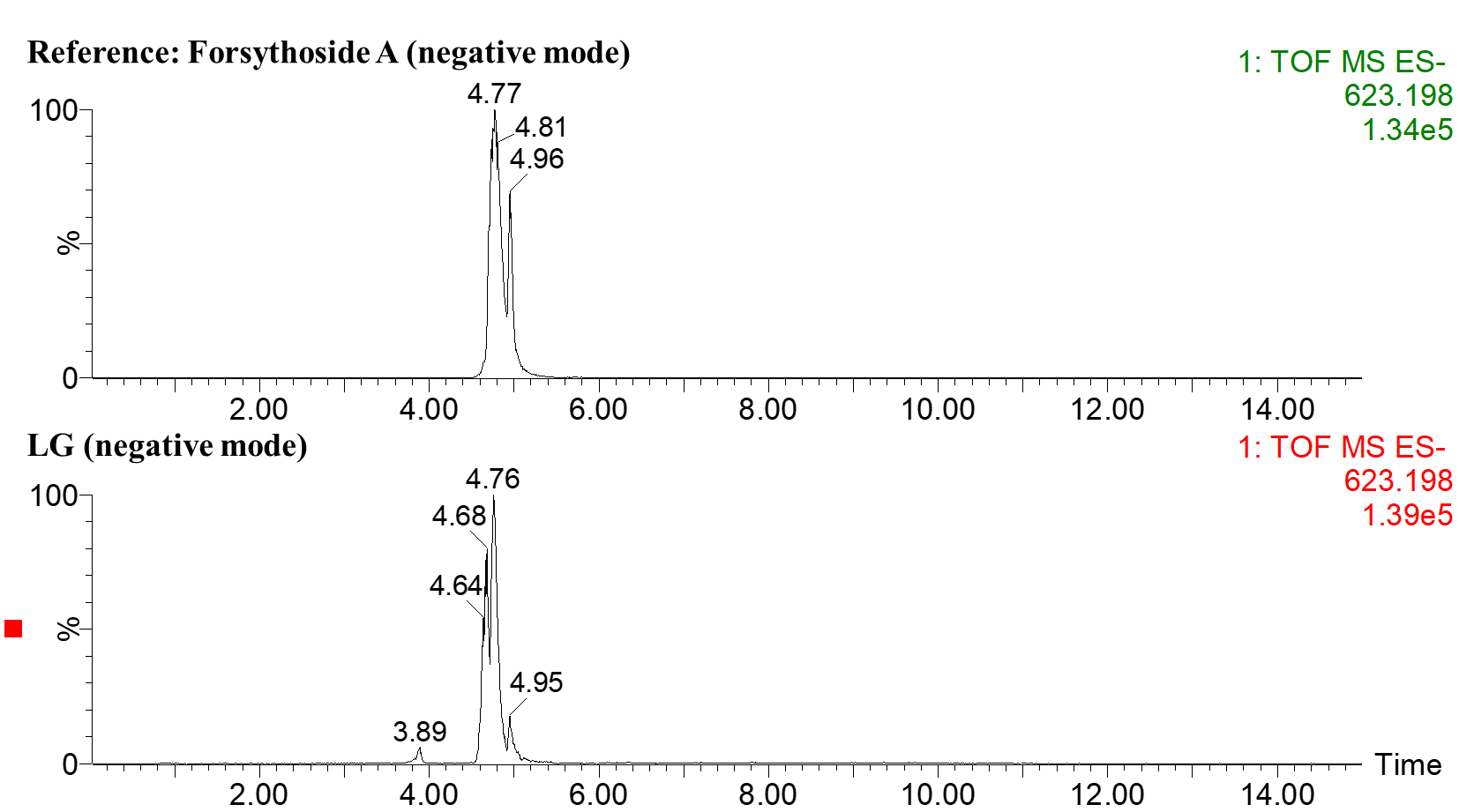


i


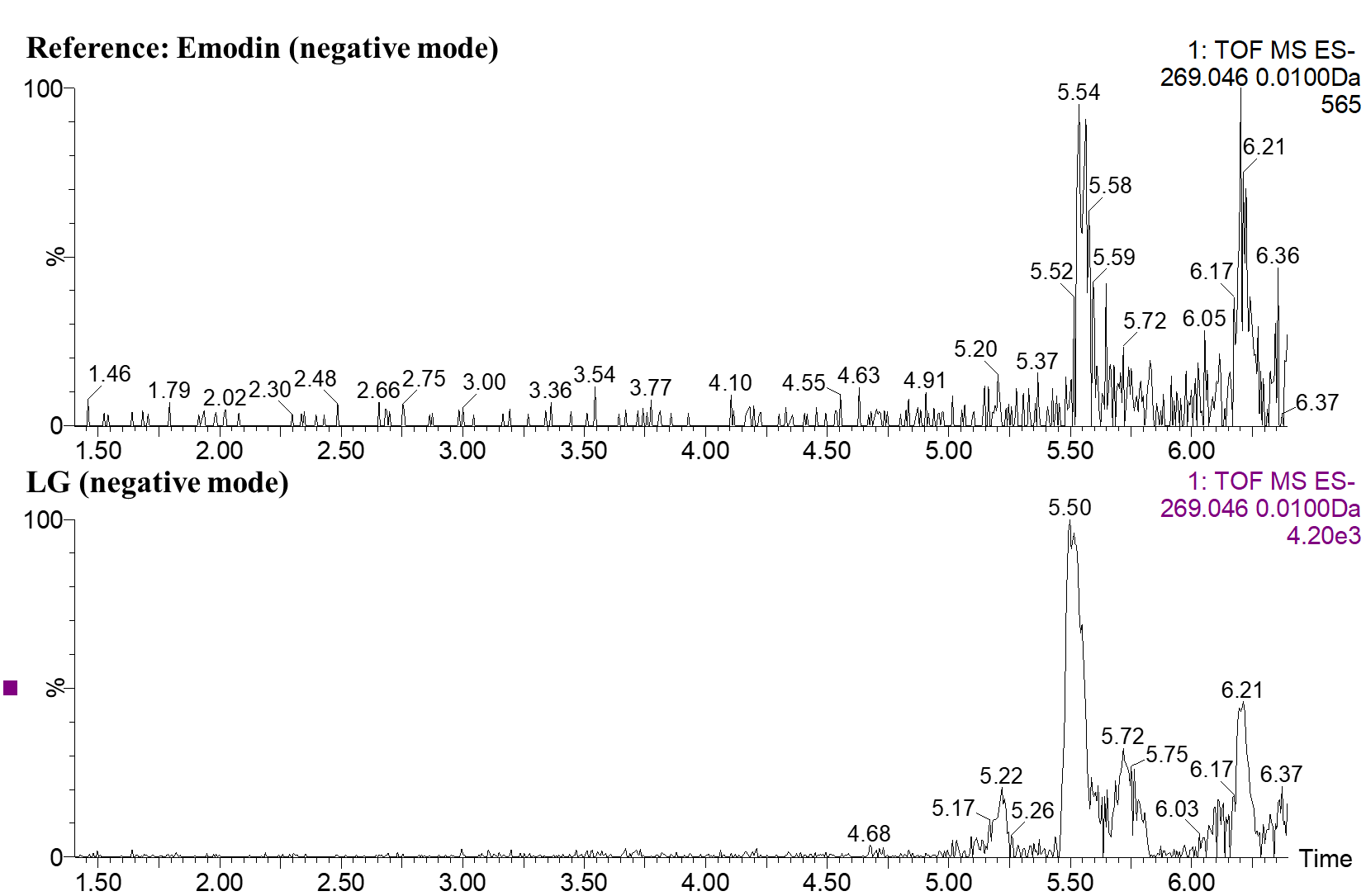


j


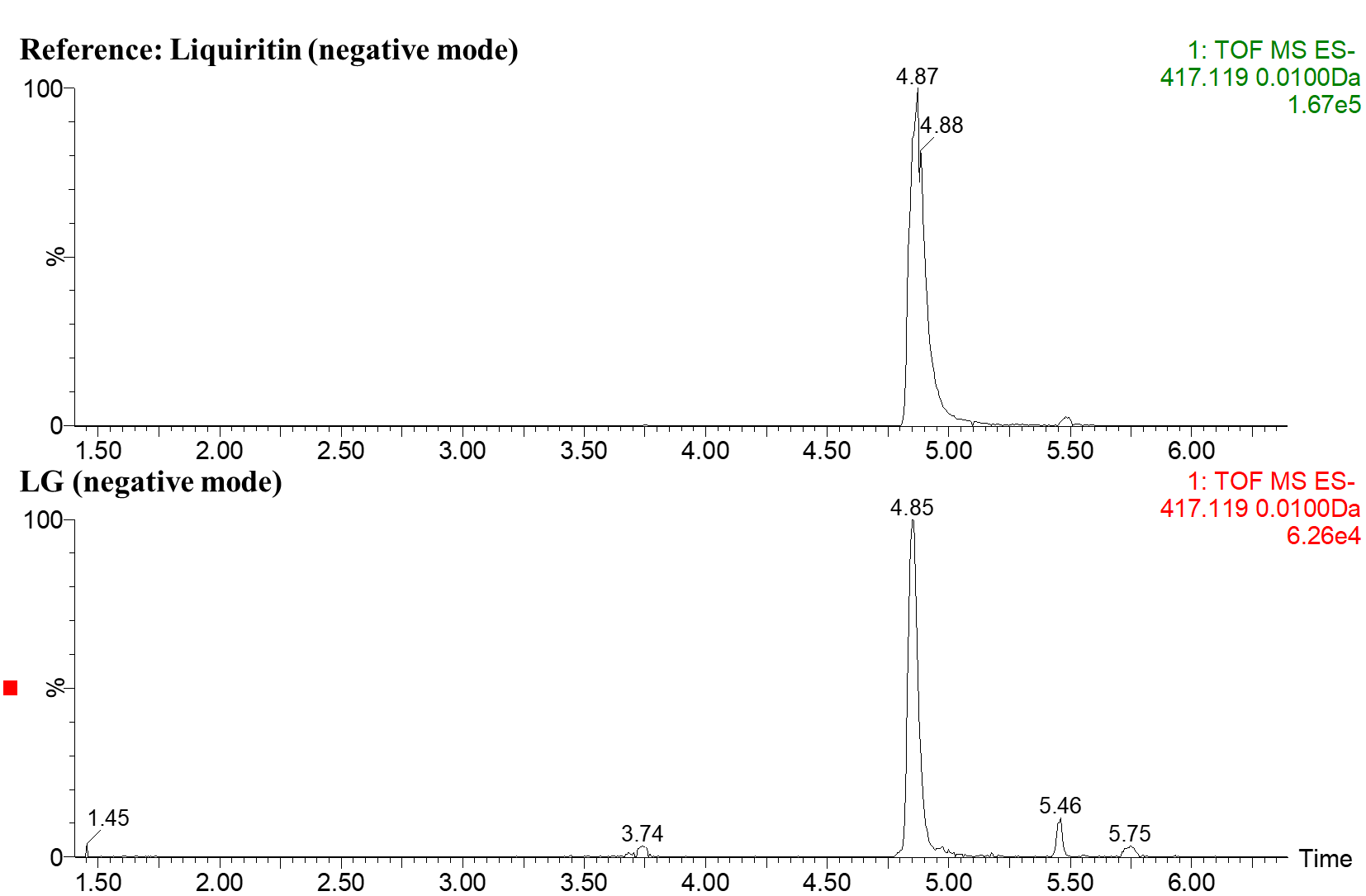


**Figure S2:** The chemical profiles of LG using UPLC-MS. (**a, b**) The total ion chromatogram in negative (**a**) and positive ion modes (**b**). (**c-t**) The main bioactive compounds of Geniposide (c), Menthol (d), Isoorientin (e), Baicalin (f), Forsythin (g), Forsythoside A (h), Emodin (i), Liquiritin (j).

**TABLE S1 The characteristic fragment ions of reference standards in LG**

| **Marking**  **peak no.** | **Name** | **RT**  **(min)** | **Ion** |
| --- | --- | --- | --- |
| 1 | Geniposide | 4.14 | [M+H]^+^ |
| 2 | Menthol | 0.77 | [M-H]^-^ |
| 3 | Isoorientin | 4.30 | [M+H]^+^ |
| 4 | Baicalin | 5.51 | [M+H]^+^ |
| 5 | Forsythin | 5.66 | [M-H]^-^ |
| 6 | Forsythoside A | 4.77 | [M-H]^-^ |
| 7 | Emodin | 5.54 | [M-H]^-^ |
| 8 | Liquiritin | 4.87 | [M-H]^-^ |
